# Chemical Profile, Antimicrobial and Anti-AChE of the Volatile Fraction of the Unexplored Bryophyte *Polytrichadelphus purpureus* Mitt. from Ecuador

**DOI:** 10.3390/plants15060980

**Published:** 2026-03-22

**Authors:** James Calva, Yamil Andrade

**Affiliations:** 1 Departamento de Química, Universidad Técnica Particular de Loja, Calle Paris s/n y Praga, Loja 110107, Ecuador; 2 Carrera de Bioquímica y Farmacia, Universidad Técnica Particular de Loja, Calle Paris s/n y Praga, Loja 110107, Ecuador; ysandrade1@utpl.edu.ec

**Keywords:** *Polytricadelphus purpureus*, (Z)-falcarinol, volatile fraction, antimicrobial, AChE

## Abstract

*Polytrichadelphus purpureus* is a bryophyte distributed in tropical and subtropical regions. It represents an underexploited source of bioactive metabolites. In this study, the volatile fraction (VF) obtained by steam distillation was analyzed by gas chromatography (GC-MS and GC-FID) on a DB-5ms capillary column, identifying 86 volatile compounds, representing the 97% of the volatile profile. Sesquiterpene hydrocarbons (23.6%), alcohols (15.6%), and alkanes (14.1%) were the major group compounds. Major components include (Z)-falcarinol (14%), hexacosane (4%), β-Curcumene (3%), and oleic acid (3%), among others. In addition, the volatile fraction exhibited moderate in vitro inhibitory activity against Gram-positive bacteria (*E. faecium*, *S. aureus*), fungus *A. niger* at concentrations of 250 µg/mL and 500 µg/mL, respectively, and *E. faecalis* and *L. monocytogenes* (250–500 µg/mL) and a weak inhibition of acetylcholinesterase (IC_50_: 392 µg/mL). These effects were evaluated for the first time in this species. While they are within the range reported for other plant-derived volatile fraction, they do not, on their own, justify claims of therapeutic efficacy. This study primarily advances our understanding of the genus Polytrichadelphus, suggesting potential as a source of bioactive sesquiterpenes for future phytochemical screening.

## 1. Introduction

Bryophytes, often overlooked as simple non-vascular plants, are emerging as a rich and underexplored source of bioactive secondary metabolites with significant pharmacological potential [1] with approximately 23,000 species described, constituting the second largest group of plants after Magnoliophyta (Angiosperms) [2,3,4]. They show low morphological complexity and a high degree of chemical diversification [5]. These ancient terrestrial organisms, which grow in humid environments as mats or cushions on soil, rocks, or vascular plants, produce a wide array of terpenoids, phenolics, and other unique compounds with documented antimicrobial, anti-inflammatory, antioxidant, and neuroprotective activities [3,5,6,7].

Recent reviews indicate that bryophytes are emerging as a promising source of volatile metabolites with complex chemical profiles as terpenes, alcohols, ketones and aromatic compounds, with compositional profiles varying across species and environment, suggesting finely regulated metabolic adaptation [6,7]. From a nutritional perspective, bryophytes lack value for human consumption, and no evidence supports their use as food [8]. Nevertheless, several species have been traditionally used in regions such as China and North America to manage fractures, snake bites, burns, upper respiratory tract infections, injuries, and certain neurological disorders, typically through decoctions, powders, or oily extracts from the plant material [9,10], further suggesting a therapeutic potential that modern science is only beginning to validate. However, the vast majority of research has focused on a few well-studied genera, leaving many species, particularly within the family Polytrichaceae, critically underexplored.

The Polytrichaceae family exhibits its greatest diversity in Southeast Asia and South America [11], comprising around 200 species and 19 genera, including *Polytrichadelphus* [12]. In Brazil, the family is represented by 6 genera and 30 species [13] while in Argentina, is represented by five genera, the Atrichum, Oligotrichum, Pogonatum, Polytrichum, and Psilopilum [14,15], distributed primarily in humid montane forests and high-altitude páramo vegetation of the Andean and sub-Andean regions. Polytrichaceae stands out for its bioactive potential, evidenced in studies of species such as *Polytrichum commune* with the presence of bornyl acetate, biformen [16], and α-pinene in *Polytrichastrum alpinum* [17]. Both profiles are associated with antioxidant, anti-inflammatory, antimicrobial, and central nervous system modulating activities, findings that suggest significant pharmacological potential for the family. Polytrichadelphus, a genus of the moss family (Polytrichaceae) comprising approximately 22 species [18] most of which are distributed in mountainous areas of South America, particularly in the Andes. Information on this genus is considered scarce, with only its descent from a Gondwanan ancestor represented by *P. magellanicus* [19]. Ecologically, these species grow on substrates on slopes, on rock and soil [20]. Although several bryophytes have documented ethnobotanical applications, no specific traditional uses have been reported for *P. purpureus*, highlighting the exploratory nature of this study.

**Figure 1:**
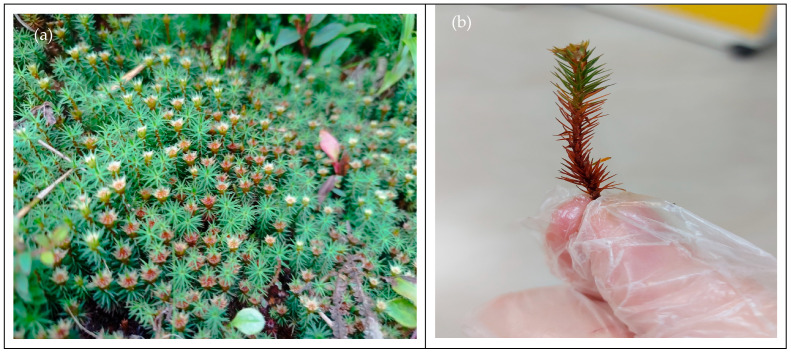
*Polytrichadelphus purpureus* of south Ecuador; (**a**) Natural State, (**b**) Individual Sample. Image provided by one of the authors (J.C.).

The present study represents the first comprehensive characterization of the volatile fraction of *P. purpureus* using gas chromatography coupled with mass spectrometry (GC-MS) and flame ionization detection (GC-FID), allowing for accurate and quantitative identification of its volatile components. In addition, the inhibitory activity on acetylcholinesterase was evaluated for the first time, as well as its antimicrobial effects against Gram-positive and Gram-negative bacteria and its antifungal activity, taking the activity of the Polytrichaceae family as a starting point. Therefore, we hypothesize that the volatile fraction of *Polytrichadelphus purpureus* (Figure 1) has a unique chemical composition, rich in bioactive terpenoids. This composition may confer moderate antioxidant and neuroprotective activities that could surpass or complement those of more commonly studied species. In a world that is always looking for natural and sustainable options, studies like this one serve two purposes. First, they expand our knowledge. Second, they broaden the range of ways in which mosses can be used in pharmacology. Mosses have traditionally been overlooked in this field, so it is great to see them receiving attention.

## 2. Results

### 2.1. Chemical Composition of Volatile Fraction

**Figure 2:**
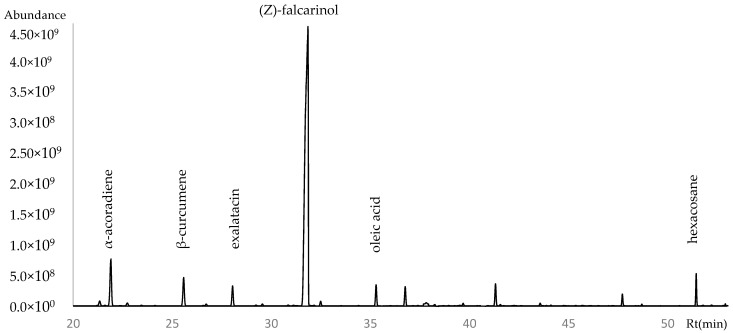
Gas chromatogram of aerial parts of *Polytrichadelphus purpureus* from Ecuador.

**Figure 3:**
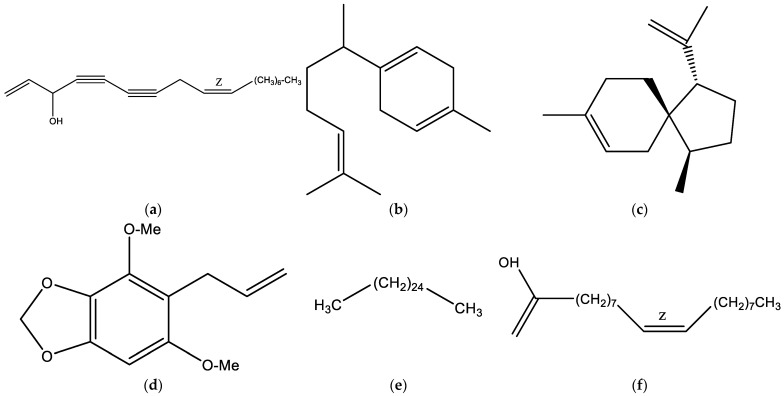
Structural formula of the major compounds present in the volatile fraction of *Polytrichadelphus purpureus* Mitt. (**a**): (Z)-Falcarinol. (**b**): β-Curcumene. (**c**): α-Acoradiene. (**d**): Exalatacin. (**e**): Hexacosane. (**f**): Oleic acid.

A total of 86 volatile compounds were identified by GC-MS, accounting for 97.35–97.55% of the total volatile content in the three samples analyzed. The chemical profile of VF from *P. purpureus* was characterized by the presence of hydrocarbon sesquiterpenes, which constituted approximately (23.6%) of the total, followed by alcohols (15.6%) and alkanes (14.1%). Among the major components were (Z)-falcarinol (14%), hexacosane (4%), oleic acid (3%), valencene (3%), α-acoradiene (3.8%), and exalatacin (3.8%). The detailed chemical composition of the VF is presented in Table 1, while the chromatogram is illustrated in Figure 2 and Figure 3.

### 2.2. Antimicrobial and Antifungal Activity

The antibacterial and antifungal activities of *P. purpureus* Mitt. VF were evaluated against reference pathogenic strains using the broth microdilution method. Ampicillin, Ciprofloxacin, and Amphotericin B were used as positive controls. The study included three Gram-positive cocci, three Gram-negative bacilli, one fungus, and one Gram-positive bacillus, respectively, as listed in Table 2. The VF exhibited moderate inhibition of the growth of *Enterococcus faecium* and *Staphylococcus aureus* at a concentration of 250 µg/mL, as well as *Aspergillus niger* at 500 µg/mL. In addition, moderate activity was observed against *Enterococcus faecalis* and *Listeria monocytogenes* at concentrations of 250 µg/mL and 500 µg/mL, respectively.

### 2.3. Inhibition of Acetylcholinesterase

**Figure 4:**
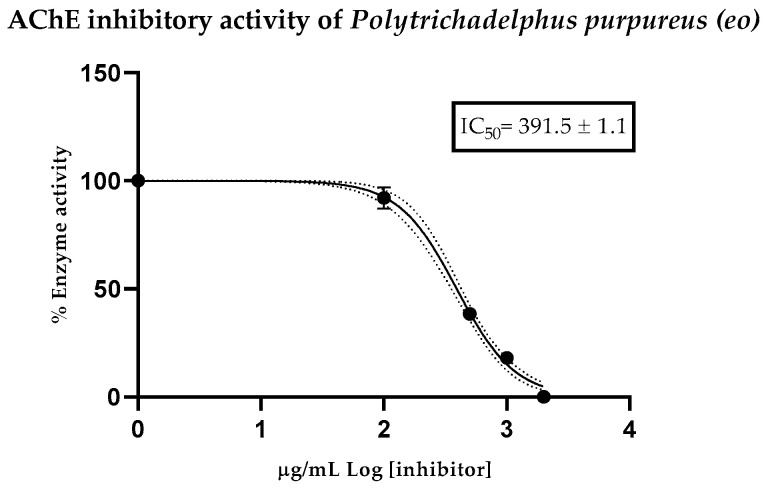
Residual cholinesterase activity curves (%) vs. concentration (μg/mL) of *Polytrichadelphus purpureus* VFs in the evaluation of anti-AChE activity.

The acetylcholinesterase enzyme was graphically represented as the logarithm of the VF concentration versus the normalized reaction response rate, allowing the determination of the mean inhibitory concentration value. VF was evaluated for the first time, obtaining an IC_50_ value of 391.5 ± 1.1 μg/mL, indicating relatively low inhibitory potency. The positive control used was Donepezil, a synthetic drug used in the treatment of dementia such as Alzheimer’s, with an IC_50_ value of 12.40 ± 1.35 μg/mL Figure 4.

## 3. Discussion

The chemical analysis of the volatile fraction from *Polytrichadelphus purpureus* Mitt., obtained by steam distillation, revealed a low yield obtained (0.08% *w*/*w*) that falls within the range previously reported for other bryophyte species, where volatile fractions rarely exceed 0.18% under hydrodistillation conditions [21,22]. This limited yield reflects the absence of specialized secretory structures such as glandular trichomes or resin canals that are characteristic of volatile-oil-producing vascular plants and enable higher accumulation of terpenoid compounds [23,24]. In contrast, volatile metabolite biosynthesis in bryophytes occurs in a more diffuse cellular manner, resulting in inherently lower concentrations of extractable volatiles [25]. The compositional profile identified 87 compounds. Our results confirm the hypothesis that this species produces an VF with a unique and highly bioactive chemical profile, dominated by sesquiterpenes and characterized by abundance of (Z)-falcarinol (~14%) followed by hexacosane, (*E*,*E*)-α-farnesene, α-acoradiene, exalatacin, valencene, oleic acid, and hydrocinnamyl acetate (2.50–3.8%). No previous reports of VF in this species have been found. However, a study of the genus by Yücel [16] reported the identification of 35 chemical compounds in the volatile fraction of *Polytrichum commune* (Hedw.) grown in Turkey, accounting for 95.48% of the total components. The major components were Biformene (13.06%), bornyl acetate (8.10%) and α-pinene (6.53%). Comparison between the VF of *P. purpureus* reported herein and the volatile fraction of *P. commune* reveals both shared and distinctive chemical features, which may reflect interspecific variation within the genus as well as geographic and ecological influences on secondary metabolite biosynthesis [26,27].

The majority compound (Z)-falcarinol is not only a significant chemical finding, but also a functional indicator of clinical interest. This polyunsaturated alkyne has been widely associated with significant biological properties, including antimicrobial activity —demonstrated by inhibition of spore germination in fungi at 20–200 μg/mL and antibacterial effects against resistant strains of Staphylococcus aureus at non-toxic concentrations [28], anti-inflammatory effects [29], antidiabetic activity through α-glucosidase inhibition [30], and antioxidant properties associated with activation of the Nrf2/HO-1 endogenous antioxidant pathway [31]. Another major compound was the hexacosane, which, according to the results of an antimicrobial test, showed moderately high activity against *Klebsiella pneumoniae*, *Salmonella typhi*, *Staphylococcus staphyloides*, and *Proteus vulgaris* [32]. Another representative compound in our oil was β-curcumene. This compound has been reported to exhibit larvicidal, antimicrobial, and pesticidal activity against Aedes, Culex, and Armigeres species [33]. Although several major constituents have well-documented biological activities, potential synergistic interactions among the multiple compounds cannot be excluded. Such interactions may significantly contribute to the overall biological activity.

Consistent with this chemical profile, the VF of *P. purpureus* exhibited selective antimicrobial activity against Gram-positive bacteria. Moderate inhibition of *E. faecium* and *S. aureus* was observed at 250 µg/mL, and moderate activity against *E. faecalis* and *L. monocytogenes* (250–500 µg/mL), with no effect on Gram-negative strains, suggesting a mechanism of action with affinity for less complex membranes, such as Gram-positive bacteria, whose cell wall lacks the lipopolysaccharide barrier characteristic of Gram-negative bacteria, whose layer limits the penetration of hydrophobic compounds, explaining the ineffectiveness of VF and wind products [34,35].

Although there are no previous studies that have directly evaluated the VF activity of *P. purpureus*, our results can be contextualized with research conducted on related species within the Polytrichaceae family. Karpiński [36] reported weak to moderate activity of extracts from *Polytrichum juniperinum* and *P. piliferum* against *E. faecalis*, *S. aureus*, and *S. pyogenes*, but not against *E. coli* or *K. pneumoniae*. The greater potency of *P. purpureus* VF compared to these extracts can be attributed to its lipophilic nature, which facilitates interaction with the lipid bilayer of Gram-positive bacterial membranes, altering their functionality and fluidity [37].

In addition, moderate antifungal activity was observed against *Aspergillus niger*, a filamentous fungus widely distributed in natural and clinical environments and responsible for food spoilage and opportunistic mycoses. Although Aruna and Krishnappa [38] had already described the antifungal activity of *Pogonatum microstomum* against *Candida albicans* and *Trichophyton rubrum*, our finding extends the spectrum of action of Polytrichaceae metabolites to filamentous fungi such as *A. niger*, suggesting a broader role in defense against microbial competitors. Although the volatile components exhibited moderate growth inhibition against Gram-positive strains, MBC assays did not distinguish its bactericidal or bacteriostatic nature. Future studies using these methods would be helpful to understand exactly how the cells are working.

Another novel finding of this study is the acetylcholinesterase (AChE) inhibitory activity of *P. purpureus* VF, with an IC_50_ value of 391.5 ± 1.1 μg/mL. While this activity is considerably lower in potency than the positive control donepezil (IC_50_ = 12.40 ± 1.35 µg/mL), it represents the first report of AChE inhibition for any species within the genus Polytrichadelphus or the Polytrichaceae family. The only precedent for anticholinesterase activity in a bryophyte volatile fraction was reported for *Syzygiella rubricaulis*, a liverwort species from Ecuador, which exhibited a considerably more potent IC_50_ of 26.75 ± 1.03 μg/mL [39]. Moderate acetylcholinesterase inhibitory activity was observed, consistent with the presence of sesquiterpenoids; however, further in vivo studies are required to assess the neuroprotective relevance. Although other bryophytes, especially liverworts, have demonstrated anti-AChE activity attributable to the presence of bibenzyls [40], terpenes including ent-longipinane-type sesquiterpenoids and labdane diterpenoids isolated from *Marsupella alpina* and *Scapania undulata*, respectively [41,42], and phenolic compounds such as flavonoids from Marchantia polymorpha [43], mosses of the Polytrichaceae family have been mainly targeted for their antimicrobial and antioxidant properties, as previously documented for *Polytrichum commune* and *Pogonatum microstomum* [44,45]. In this context, our findings not only fill a critical knowledge gap in bryophyte chemistry but also establish *P. purpureus* represents a novel chemosystematic record for bryophytes, with potential for further exploration in natural product chemistry.

## 4. Materials and Methods

### 4.1. Plant Collection

The aerial parts of *P. purpureus* were collected in the sector “El Tiro”, on the provincial border between Loja and Zamora provinces in southern Ecuador, at an altitude of 2372 m. a.s.l. The latitude was 3°59′10″ S and the longitude was 79°10′23″ W. It was collected in May-July 2024 under the following climatic conditions estimated using data from the WorldClim v2.1 global climate database [46] mean air temperature of 17 °C, with a maximum of 21 °C and a minimum of 14 °C; average relative humidity of approximately 82%; mean wind speed of 1.8 m s^−1^; and a mean monthly precipitation of around 80 mm. These values represent the typical climatic conditions for the Andean montane environment of southern Ecuador during the wet-to-dry seasonal transition. All specimens were collected from the same specific community to ensure uniformity in environmental growth conditions and population source. The specimen was identified by Jorge Luis Armijos, a botanist at the Herbarium and Museum of Biological Collections of the UTPL. A voucher specimen has been deposited at the Herbarium of the Universidad Técnica Particular de Loja (UTPL).

### 4.2. Extraction of Volatile Compounds

The collected plant material was divided into three subsamples per collection and distilled using fresh material immediately after harvesting. Before extraction, the material was finely chopped to increase the surface area and improve the efficiency of the distillation process. The VF was extracted by steam distillation using a modified Dean-Stark apparatus and the procedure described by Jaramillo S.P. et al. [47]. The extraction was performed in three individual systems. In each unit, c.a 200 g of plant material was introduced and subjected to a continuous 4 h process at a temperature of approximately 100 °C. The VF was transferred to 2 mL amber bottles to preserve its physicochemical properties through refrigerated storage at −7 °C. It should be noted that this process was carried out in triplicate to ensure reproducibility and statistical reliability. Finally, the yield was determined as percent mass (g) of volatile fraction per mass (g) of plant material (% *w*/*w*) [48].

### 4.3. Chemical Composition of the Volatile Components

In order to determine the chemical composition of the VF, chemical analysis was performed using gas chromatography coupled with mass spectrometry (GC-MS), supplemented by flame ionization detection (GC/FID).

### 4.4. Qualitative Analysis

The chemical composition of the VF was performed using a Thermo Fisher Scientific Trace 1310 series 7200002174 gas chromatograph (Waltham, MA, USA) coupled with a Thermo Fisher Scientific ISQ 7000 mass spectrophotometer (GC-MS). For this purpose, three 1 μL volumes of a VF solution dissolved in hexane were injected in split ratio mode (40:1) into the non-polar DB5-ms capillary column (5% phenyl-methylpolysiloxane, 30 m × 0.25 mm × 0.25 μm). The column temperature was set at 50 °C with an increase of 3 °C/minute until reaching 230 °C. Helium was used as the carrier gas (1 mL/min) with an initial pressure of 6.49 psi and an average pressure of 35 cm/s [49].

The compounds were identified by comparing the mass spectra tentatively assigned based on GC-MS fragmentation patterns and the calculated linear retention indices (LRI) described in the literature NIST [50] and Adams 2007 [51]. A value of ±20 units was considered acceptable. The LRI was calculated using the method of Van Den Dool and Kratz [52], employing a mixture of n-alkanes (C10-C25) injected under chromatographic conditions of the VFs.

### 4.5. Quantitative Analysis

The compounds were determined using the same Thermo Fisher Scientific Trace 1310 gas chromatograph, which had a flame ionization detector (GC-FID). 1 µL of the volatile fraction was dissolved (1:100) in hexane solution and injected into the DB5-ms capillary column using helium as the carrier gas. For FID detection, a hydrogen-air gas mixture was used to quantify the ions produced by the combustion of the different compounds present in the VF of interest. Percentages were calculated by the peak area normalization method using GC-FID data.

### 4.6. Antimicrobial Activity

Antimicrobial activity was measured using broth microdilution techniques, following the methodology described by Cartuche et al. [53]. The activity was evaluated using bacterial strains from the American Type Culture Collection (ATCC). Gram-positive microorganisms (*E. faecalis* ATCC ^®^ 19433, *E. faecium* ATCC ^®^ 27270, *S. aureus* ATCC ^®^ 25923, *L. monocytogenes* ATTC ^®^ 19115) and Gram-negative microorganisms (*E. coli* (O157:H7) ATCC^®^ 43888, *P. aeruginosa* ATCC^®^ 10145, *S. enterica Typhimurium* WDCM 00031, derived ATCC^®^ 14028) and the fungus *Aspergillus niger* ATCC^®^ 6275. The bacteria were cultured in Mueller-Hinton medium for 24 h at a temperature of 37 °C and the fungus in Sabouraud broth at a temperature of 28–30 °C for 72 h.

Due to the hydrophobic nature of volatile fractions, dimethyl sulfoxide (DMSO) was used as a cosolvent to ensure complete solubility in the aqueous broth medium. The VF was prepared at a concentration of 10 mg/mL in 1% DMSO. Dilutions were performed in concentrations of 250 µg/mL to 500 µg/mL. Microbial suspensions were adjusted to 0.5 McFarland standard (approx. 1.5 × 10^8^ CFU/mL) and subsequently diluted 1:100. Then, 100 µL of the VF and 100 µL of the microbial suspension were added to each microdilution. A negative control of 5% DMSO and positive controls of specific antibiotics were used: ciprofloxacin (1 mg/mL) and ampicillin (1 g/mL) for bacteria and erythromycin (1 mg/mL) and amphotericin B (250 µg/mL) for fungi. Antimicrobial activity was evaluated by turbidity in bacteria and mycelial growth in fungi. After incubation, bacterial growth was assessed by measuring optical density (OD) at 600 nm using a microplate reader EPOCH 2 (BioTek, Santa Clara, CA, USA). In the case of fungi, resazurin was used as a color indicator of viability (blue to pink). All assays were performed in triplicate (*n* = 3), and results are reported as mean values with standard deviations.

### 4.7. Acetylcholinesterase Activity

The AChE inhibitory capacity of VF from *P. purpureus* was evaluated following the method described by Chouit et al. [54], with modifications applied by Cartuche et al. [53]. The reaction mixture was prepared using a Tris buffer solution (pH 8.0), acetylcholine (ATCh, 15 mM in PBS, pH 7.4), DTNB (3 mM in Tris), and the volatile fraction sample. After pre-incubation for three minutes at 25 °C with constant stirring, acetylcholinesterase (0.5 U/mL) was added to initiate the reaction. Product release was measured at 405 nm using an EPOCH 2 microplate reader (BioTek) over 60 nm. Due to the hydrophobic nature of the VF, methanol was employed as a cosolvent to ensure initial solubility; the VF was prepared as a stock solution at 10 mg/mL in methanol and subsequently diluted to achieve final assay concentrations of 1000, 500, 100, 50, and 10 µg/mL. The final methanol concentration in the reaction mixture did not exceed 10% (*v*/*v*), a level previously verified to have no significant effect on AChE activity. To account for potential turbidity or background absorbance, sample blanks (containing all reagents except the enzyme) were included for each concentration, and their absorbance values were subtracted from the corresponding reaction wells. The reaction rate was determined using a calibration curve with DTNB and L-GSH at various concentrations. Methanol (10% *v*/*v*) served as the negative control, and donepezil hydrochloride was used as a positive control (IC_50_ = 12.40 ± 1.35 µM). All assays were performed in triplicate (*n* = 3).

Precise structure-activity correlations require further investigation due to the possibility of synergistic interactions. Future bioassay-guided fractionation is necessary to isolate the active constituents and determine their specific contributions.

## 5. Conclusions

This study is the first report on the chemical composition and biological activities of the volatile fraction of *P. purpureus*, a neotropical moss previously unexplored phytochemically and pharmacologically. Eighty-six volatile compounds were identified, notably (Z)-falcarinol as the major component (14%). The VF showed a moderate antimicrobial activity against Gram-positive pathogens (*Enterococcus faecium*, *Staphylococcus aureus*) and the fungus *Aspergillus niger*. In addition, moderate to low AChE inhibitory activity (IC_50_ = 392 µg/mL) was reported for the first time in the Polytrichaceae family, expanding its bioactive profile beyond antimicrobial activity. Collectively, these findings position *P. purpureus* VF as a promising and previously untapped source of natural metabolites with potential relevance for human health. They also highlight the potential of bryophytes as an under-explored source of chemical diversity. This work establishes a basis for future studies to optimize its bioactive constituents, elucidate their mechanisms of action, and advance the sustainable utilization of neotropical mosses in drug discovery.

## Figures and Tables

**Table 1:** Chemical analysis of the volatile fraction of *Polytrichadelphus purpureus* using a nonpolar stationary phase column.

				5% Phenyl, 95% Dimethylpolysiloxane			
N°	Compound	LRI ^a^	LRI ^b^	May	June	July	M.F.
				% ± SD			
1	hexenol	849	844	0.37 ± 0.05	0.38 ± 0.03	0.36 ± 0.05	C_6_H_12_O
2	tricyclene	934	921	0.37 ± 0.09	n.d.	n.d.	C_10_H_16_
3	tolualdehyde	1062	1062	0.68 ± 0.05	0.64 ± 0.08	0.7 ± 0.10	C_8_H_8_O
4	nonanal	1117	1100	0.77 ± 0.02	0.79 ± 0.03	0.74 ± 0.09	C_9_H_18_O
5	n-decanal	1218	1201	0.68 ± 0.07	0.66 ± 0.08	0.64 ± 0.07	C_10_H_20_O
6	carvenone	1250	1255	0.46 ± 0.05	0.47 ± 0.05	0.4 ± 0.04	C_10_H_16_O
7	(2E)-decenal	1276	1260	0.39 ± 0.06	0.39 ± 0.0	0.35 ± 0.10	C_10_H_18_O
8	Iso-menthyl acetate	1319	1304	0.46 ± 0.12	0.46 ± 0.11	0.45 ± 0.08	C_12_H_22_O_2_
9	(2E)-undecenal	1354	1357	0.49 ± 0.05	0.50 ± 0.06	0.46 ± 0.10	C_11_H_20_O
10	hydrocinnamyl acetate	1366	1366	2.66 ± 0.09	2.57 ± 0.10	2.4 ± 0.12	C_11_H_14_O_2_
11	2E-octenol butanoate	1392	1386	n.d.	n.d.	0.34 ± 0.012	C_12_H_22_O_2_
12	α-chamipinene	1396	1396	0.51 ± 0.05	0.49 ± 0.15	0.50 ± 0.10	C_15_H_24_
13	dihidro-α-Ionone	1411	1411	0.67 ± 0.02	0.64 ± 0.05	0.67 ± 0.0	C_13_H_22_O
14	α-santalene	1416	1416	2.03 ± 0.05	1.96 ± 0.014	1.89 ± 0.09	C_15_H_24_
15	4,8-β-epoxy-caryophyllane	1421	1423	0.88 ± 0.07	0.86 ± 0.03	0.81 ± 0.05	C_15_H_26_O
16	α-himachalene	1440	1449	0.46 ± 0.05	0.46 ± 0.01	0.42 ± 0.09	C_15_H_24_
17	α-acoradiene	1452	1464	3.91 ± 0.05	3.83 ± 0.06	3.61 ± 0.03	C_15_H_24_
18	(*E*)-β-farnesene	1457	1454	1.04 ± 0.10	1.29 ± 0.05	0.96 ± 0.08	C_15_H_24_
19	cumacrene	1459	1470	0.58 ± 0.05	0.26 ± 0.07	0.5 ± 0.0	C_15_H_24_
20	γ-muurolene	1478	1478	n.d.	0.09 ± 0.08	n.d.	C_15_H_24_
21	cumacrene	1481	1470	0.29 ± 0.13	0.37 ± 0.02	0.27 ± 0.05	C_15_H_24_
22	γ-curcumene	1485	1481	1.43 ± 0.05	1.36 ± 0.01	1.31 ± 0.09	C_15_H_24_
23	cis-Eudesma-6, 11-diene	1487	1489	0.76 ± 0.05	0.70 ± 0.09	0.71 ± 0.05	C_15_H_24_
24	cuparene	1494	1504	0.86 ± 0.08	0.82 ± 0.02	0.76 ± 0.07	C_15_H_22_
25	γ-amorphene	1497	1495	1.36 ± 0.05	1.36 ± 0.10	1.26 ± 0.10	C_15_H_24_
26	valencene	1499	1496	3.01 ± 0.15	2.93 ± 0.12	2.77 ± 0.16	C_15_H_24_
27	β-macrocarpene	1505	1499	0.71 ± 0.05	0.70 ± 0.01	0.64 ± 0.05	C_15_H_24_
28	(*E*, *E*)-α-farnesene	1515	1505	3.97 ± 0.05	3.86 ± 0.06	3.59 ± 0.06	C_15_H_24_
29	β-curcumene	1523	1514	0.39 ± 0.02	0.41 ± 0.08	0.38 ± 0.09	C_15_H_24_
30	δ-cadinene	1527	1522	1.25 ± 0.09	1.19 ± 0.09	1.10 ± 0.07	C_15_H_24_
31	β-bazzanene	1531	1519	0.35 ± 0.05	0.53 ± 0.03	0.49 ± 0.01	C_15_H_24_
32	γ-cuprenene	1536	1532	n.d.	0.21 ± 0.12	0.18 ± 0.02	C_15_H_24_
33	trans-dauca-4(11),7-diene	1546	1556	1.03 ± 0.05	0.99 ± 0.05	0.93 ± 0.04	C_15_H_24_
34	globulol	1577	1590	0.84 ± 0.03	0.81 ± 0.0	0.78 ± 0.09	C_15_H_26_O
35	β-copaen-4-α-ol	1586	1590	0.62 ± 0.04	0.57 ± 0.06	0.7 ± 0.05	C_15_H_24_O
36	spathulenol	1590	1577	0.02 ± 0.01	n.d.	0.02 ± 0.01	C_15_H_24_O
37	salvial-4(14)-en-1-one	1595	1594	1.69 ± 0.05	1.69 ± 0.0	1.6 ± 0.15	C_15_H_24_O
38	hexadecane	1601	1600	0.02 ± 0.03	n.d.	0.01 ± 0.02	C_16_H_34_
39	khusimone	1604	1604	0.52 ± 0.03	0.43 ± 0.09	0.47 ± 0.12	C_14_H_20_O
40	anti-anti-anti-helifolen-12-al B	1608	1592	0.01 ± 0.05	n.d.	0.01 ± 0.02	C_15_H_22_O
41	β-cedrene epoxide	1624	1621	0.43 ± 0.02	0.41 ± 0.09	0.4 ± 0.05	C_15_H_24_O
42	α-corocalene	1628	1622	0.61 ± 0.12	0.60 ± 0.07	0.57 ± 0.05	C_15_H_20_
43	α-muurolol (=Torreyol)	1644	1644	0.93 ± 0.10	0.87 ± 0.02	0.85 ± 0.08	C_15_H_26_O
44	selina-3,11-dien-6-α-ol	1646	1642	1.53 ± 0.16	1.62 ± 0.10	1.45 ± 0.12	C_15_H_24_O
45	valerianol	1650	1656	0.60 ± 0.02	0.49 ± 0.05	0.56 ± 0.02	C_15_H_26_O
46	exalatacin	1662	1655	3.85 ± 0.09	3.80 ± 0.11	3.6 ± 0.16	C_12_H_14_O_4_
47	α-bisabolol	1667	1685	0.63 ± 0.05	0.63 ± 0.07	0.61 ± 0.08	C_15_H_26_O
48	elemol acetate	1671	1680	0.42 ± 0.05	0.42 ± 0.04	0.39 ± 0.08	C_17_H_28_O_2_
49	n-tetradecanol	1680	1671	0.93 ± 0.05	0.89 ± 0.07	0.85 ± 0.10	C_14_H_30_O
50	germacra-4(15), 5, 10(14)-trien-1-α-ol	1686	1685	0.01 ± 0.05	n.d.	n.d.	C_15_H_24_O
51	sesquicineol-2-one	1688	1702	0.52 ± 0.02	0.47 ± 0.12	0.5 ± 0.07	C_15_H_24_O_2_
52	n-heptadecane	1701	1700	1.85 ± 0.08	1.85 ± 0.12	1.72 ± 0.14	C_17_H_36_
53	(2E)-tridecenol acetate	1726	1703	2.62 ± 0.05	2.53 ± 0.09	2.41 ± 0.02	C_15_H_28_O_2_
54	cedr-8(15)-en-9-α-ol, acetate	1734	1741	0.71 ± 0.18	0.69 ± 0.13	0.65 ± 0.15	C_17_H_26_O_2_
55	ethyl tetradecanoate	1783	1795	0.80 ± 0.05	1.12 ± 0.12	1.08 ± 0.11	C_16_H_32_O_2_
56	1-octadecene	1790	1789	n.d.	0.72 ± 0.05	0.71 ± 0.08	C_18_H_36_
57	cyclopentadecanolide	1838	1832	0.56 ± 0.16	0.56 ± 0.15	0.53 ± 0.17	C_15_H_28_O_2_
58	not identified	1859	n.d.	2.46 ± 0.12	2.46 ± 0.08	2.30 ± 0.05	n.d.
59	n-hexadecanol	1897	1874	n.d.	n.d.	0.56 ± 0.03	C_16_H_32_O
60	8S,13-cedranediol	1901	1897	0.75 ± 0.02	0.72 ± 0.09	0.79 ± 0.03	C_15_H_26_O_2_
61	rimuene	1908	1896	0.88 ± 0.11	0.79 ± 0.09	0.75 ± 0.05	C_20_H_32_
62	dihydro-columellarin	1911	1900	0.03 ± 0.05	n.d.	n.d.	C_15_H_22_O_2_
63	n-nonadecane	1915	1900	0.47 ± 0.13	n.d.	n.d.	C_19_H_40_
64	cyclohexadecanolide	1931	1933	0.79 ± 0.11	0.80 ± 0.07	0.74 ± 0.15	C_16_H_30_O_2_
65	musk ambrette	1936	1929	0.62 ± 0.17	0.62 ± 0.05	0.57 ± 0.09	C_16_H_28_O_2_
66	hexadecyl acetate	1986	2003	2.23 ± 0.16	2.86 ± 0.10	2.75 ± 0.12	C_18_H_36_O_2_
67	Not identified	1998	n.d.	n.d.	0.19 ± 0.08	0.19 ± 0.05	n.d.
68	1-eicosene	2005	1987	0.70 ± 0.12	0.79 ± 0.06	0.76 ± 0.04	C_20_H_40_
69	ethylene brassylate	2032	2015	n.d.	0.21 ± 0.01	n.d.	C_15_H_26_O_4_
70	(6Z, 10E)-pseudo phytol	2039	2030	0.77 ± 0.05	0.79 ± 0.06	0.7 ± 0.10	C_20_H_36_O
71	(Z)-falcarinol	2050	2035	14.35 ± 0.19	14.33 ± 0.12	13.47 ± 0.09	C_17_H_24_O
72	kaurene	2057	2042	2.17 ± 0.21	2.19 ± 0.10	2.05 ± 0.15	C_20_H_32_
73	n-heneicosane	2103	2100	1.32 ± 0.02	1.33 ± 0.08	1.24 ± 0.07	C_21_H_44_
74	methyl linoleate	2113	2095	0.04 ± 0.05	0.04 ± 0.08	0.04 ± 0.03	C_19_H_34_O_2_
75	laurenan-2-one	2118	2115	2.42 ± 0.22	2.42 ± 0.18	2.28 ± 0.12	C_20_H_32_O
76	nezukol	2122	2132	1.20 ± 0.19	1.21 ± 0.08	1.12 ± 0.03	C_20_H_34_O
77	oleic acid	2154	2141	2.94 ± 0.06	2.98 ± 0.04	2.79 ± 0.08	C_18_H_34_O_2_
78	abineol	2158	2149	0.73 ± 0.05	0.75 ± 0.06	0.72 ± 0.10	C_20_H_34_O
79	1-docosene	2199	2189	0.62 ± 0.09	0.28 ± 0.07	0.28 ± 0.09	C_23_H_44_
80	n-docosane	2203	2200	0.75 ± 0.10	1.08 ± 0.10	1.02 ± 0.08	C_23_H_46_
81	methyl eperuate	2239	2220	n.d.	n.d.	0.34 ± 0.07	C_21_H_36_O_2_
82	n-tricosane	2302	2300	2.52 ± 0.05	2.78 ± 0.02	2.59 ± 0.04	C_23_H_48_
83	4-epi-abietol	2350	2343	0.41 ± 0.03	n.d.	n.d.	C_20_H_32_O
84	n-tetracosane	2427	2400	1.47 ± 0.08	1.50 ± 0.10	1.34 ± 0.15	C_24_H_50_
85	n-pentacosane	2492	2500	1.69 ± 0.05	2.92 ± 0.09	2.53 ± 0.12	C_25_H_52_
86	hexacosane	2590	2600	4.12 ± 0.13	2.62 ± 0.12	2.47 ± 0.13	C_26_H_54_
	Total identified (%)			97.55	97.35	97.39	
	Sesquiterpene hydrocarbons (%)			23.94	23.84	23.33	
	Oxygenated sesquiterpene (%)			13.29	13.03	13.19	
	Hydrocarbon monoterpene (%)			0.37	0	0	
	Oxygenated monoterpene (%)			1.53	1.52	1.46	
	Oxygenated diterpenes (%)			5.53	5.17	5.05	
	Hydrocarbon diterpenes (%)			3.76	3.76	3.73	
	Alkanes (%)			14.21	14.07	13.54	
	Alcohols (%)			15.65	15.59	15.97	
	Aldehydes(%)			1.94	1.93	1.99	
	Alkenes (%)			0	0.72	0.74	
	Esters (%)			7.32	8.16	8.84	
	Other			10.01	9.55	9.55	
	Not identified			2.46	2.65	2.61	

LRI ^a^ Calculated linear retention index; LRI ^b^ Linear retention index according to literature; % ± SD percentage with standard deviation; mean ± SD; n.d. = not detectable; values are the mean of three determinations (*n* = 3); M.F. = molecular formula.

**Table 2:** Minimum inhibitory concentration (mg/mL) of VF from *Polytrichadelphus purpureus* on microorganisms that cause infections.

Microorganisms	*Enterococcus faecalis* ATCC ^®^ 19433	*Enterococcus faecium* ATCC ^®^ 27270	*Staphylococcus aureus* ATCC ^®^ 25923	*Escherichia coli (O157:H7)* ATCC ^®^ 43888	*Pseudomonas aeruginosa* ATCC ^®^ 10145	*Salmonella enterica* subs enterica serovar Thypimurium WDCM 00031, derived ATCC ^®^ 14028	*Aspergillus niger* ATCC ^®^ 6275	*Lysteria monocytogenes* ATTC ^®^ 19115
*Polytrichadelphus purpureus*	250	250	250	-	-	-	500	250
Ampicillin (1 g/mL)	0.7812	<0.3906	<0.3906	-	-	-	-	-
Ciprofloxacin (1 mg/mL)	-	-	-	15.625	<0.3906	<0.3906	-	15.625
Amphotericin B (250 µg/mL)	-	-	-	-	-	-	<0.098	-
Dimethylsulfoxide 5%	+	+	+	+	+	+	+	+

(-) Not active at the highest tested dose of 500 µg/mL, (+) Normal growth at 5% DMSO.

## Data Availability

The original contributions presented in the study are included in the article, which provides a comprehensive overview of the research findings. Further enquiries should be directed to the corresponding author.

## Abbreviations

The following abbreviations are used in this manuscript:

VF	Volatile Fraction
GC-MS	Gas Chromatography- Mass Spectrum
GC-FID	Gas Chromatography-Flame Ionization Detector
AChE	Acetylcholinesterase
ATCC	American Type Culture Collection
